# Seasonal Change in Activity Rhythms and Time Budgets of Tibetan Macaques

**DOI:** 10.3390/biology11091260

**Published:** 2022-08-24

**Authors:** Jie Zhou, Wen-Bo Li, Xi Wang, Jin-Hua Li

**Affiliations:** 1 School of Resources and Environmental Engineering, Anhui University, Hefei 230601, China; 2 International Collaborative Research Center for Huangshan Biodiversity and Tibetan Macaque Behavioral Ecology, Hefei 230601, China; 3 Key Laboratory of Animal Ecology and Conservation Biology, Institute of Zoology, Chinese Academy of Sciences, Beijing 100101, China; 4 School of Life Sciences, Hefei Normal University, Hefei 230601, China

**Keywords:** activity rhythm, time budget, Tibetan macaques (*Macaca thibetana*), temperature and food factors

## Abstract

**Simple Summary:**

Primate activity rhythms and time budgets are dictated by food availability, temperature, and social factor; thus, primates must adapt their behaviors to accommodate fluctuations in environmental change. We conducted a year-long field study in the Huangshan region to collect data on Tibetan macaques’ behavior and local weather. We compiled and analyzed these data, explored Tibetan macaques’ behavioral patterns, and analyzed the potential factors that differentiate these behavioral patterns. The results indicate that Tibetan macaques have two foraging peaks and one resting peak in a day. Temperature and food were the main factors affecting the activity time budget of Tibetan macaques. Tibetan macaques’ daily activity time budgets are significantly different in sex and age group.

**Abstract:**

Activity rhythms and time budgets are important to understand behavioral variability and adaptation in primates because animals normally use a behavioral adjustment as a preferential choice in response to environmental changes. Therefore, we observed a group of un-provisioned Tibetan macaques (*Macaca thibetana*) in Tianhu Mountain County Nature Reserve, Mount Huangshan, Southern Anhui, China. We used the instantaneous scan sampling method to collect behavioral data on their activity rhythms and time budget. The results showed that Tibetan macaques have two foraging peaks (9:00–10:00 and 14:00–15:00) and a resting peak at 12:00–13:00. They spent 31.96% resting, 28.59% foraging, 26.96% moving, 6.90% grooming, and 5.59% other. The total time of foraging and moving per month significantly and positively correlated with fruits and buds and negatively correlated with leaves. Different sexes and age groups demonstrated different activity time budgets, with adult males, adult females, and young macaques spending most of their time resting, grooming, and playing, respectively. We elucidated the effects of different environmental conditions on Tibetan macaques and their behavioral adaptation strategies.

## 1. Introduction

Living in groups reduces predation risk for animals, but it increases intra-group competition for limited resources [1,2]. Thus, animals must spend extra time foraging or finding new foraging sites daily to compensate for foraging competition [3,4]. Many non-human primates, such as Rhesus macaques (*Macaca mulatta*), spend more than a third of their day foraging. Furthermore, they have to invest many times in establishing and maintaining social relationships [5], as these activities affect their possession of food and matting mates [6]. Activity rhythms and time budgets are two essential aspects of primates that are directly related to their metabolic and energetic limitations. These conditions, in turn, change in response to different environments [7].

Activity rhythms refer to several behaviors that animals are required to perform every day, usually some relatively fixed behavioral patterns [8]. The daily activity rhythms of many primates have been studied, and the results demonstrate that the vast majority of primates have two foraging peaks, one in the morning and the other in the afternoon, and a resting peak at midday (e.g., white-headed langur (*Trachypithecus leucocephalus*), Francois’ langur (*Trachypithecus francoisi*), and (*M. mulatta*)) [9,10,11,12]. The main factors influencing the daily activity rhythm are classified as follows: changes in the climate, competition between species, predation risk, and anthropogenic disturbance [13]. One of the most important factors is the climatic conditions change, which can be classified as temperature, light, relative humidity, etc. [8]. For example, Francois’ langurs’ activity rhythms are affected by temperature and light. In summer, they choose to hide in the woods or caves for rest. However, they sit on rocks in winter to bask in the sun [5]. Therefore, environmental factors greatly influence the activity rhythm of primates.

Activity budget refers to the phenomenon wherein animals divide their time for various daily activities according to their basic ecological needs, such as food, matingmates, and rest [14]. Numerous studies have reported that various ecological factors affect activity time budgets in primates, such as climatic conditions [15], food availability, and distribution [16,17]. Food resources play an essential role in the primates’ time budget [18]. In general, leaves are low-quality food compared with fruits. Frugivorous primates do not allocate much time foraging and rest more than frugivorous primates [19,20]. For example, Francois’ langurs spend most of their time resting, with up to 51.5% of their rest time spent effectively fermenting and breaking down fiber-rich leaves, which make up 52.8% of their diet, whereas fruits and seeds account for only 31.4% [21,22]. Furthermore, primates can flexibly adjust their activity time budgets according to the different seasons. Japanese macaques (*M. fuscata*) live at high latitudes with a scarcity of high-quality food in winter. Therefore, they increase their rest and social time and decrease their foraging time [15]. However, Japanese macaques’ activity time budget does not apply to other primates. For example, white-headed langurs living in karst rocky mountains adopt the opposite adjustment strategy, increasing their foraging and movement time and reducing their resting time in winter when food is relatively scarce [10]. Therefore, food resources are the main factor affecting the primates’ activity time budgets.

Tibetan macaques (*M. thibetana*) or cercopithecidae belong to the order Primates, and genus *Macaca* is classified as Near Threatened (IUCN 2020) and endemic to east-central and south China [23]. Its distribution is almost entirely north of the Tropic of Cancer. The climate type is temperate and warm subtropical, which is not rare for >200 species of primates worldwide. Wang et al. (2008) studied a group of Tibetan macaques in Huangshan Mountain [24]. The results showed that their main activity was resting (59.72%), followed by grooming (17.50%), foraging (14.82%), wandering (5.86%), and playing (0.04%), and others (2.06%). The peak time of foraging coincided with the time of artificial food provision. However, the drawback of this study is that artificial food affects Tibetan macaques’ activity time budget. The results failed to show the real activity time budget of Tibetan macaques in the un-provisioned. Liu et al. (2020) investigated daily and seasonal variation of activities of Tibetan macaques based on infrared camera trapping [25]. The results demonstrated that Tibetan macaques are typical diurnal animals. The daily activity showed an “M” pattern, with peak activity occurring between 12:00 and 16:00. Tibetan macaque’s activity time is shorter during the dry season than in the rainy season. Their main activity time is delayed from noon to afternoon. However, the drawback of this study is that the sample size was too small. Tibetan macaques are widely distributed in the mountainous areas of southern Anhui Province with little human interference. In-depth knowledge and systematic long-term quantitative studies on their activity rhythms and time budgets are lacking.

In this study, We conducted an un-provisioned group of Tibetan macaques in Tianhu Mountain County Nature Reserve, Huangshan District, Southern Anhui Province, to understand their activity rhythms and time budgets comprehensively. Taking activity rhythm and time budgets as the starting point, we analyze the relationship between activity rhythm and time budgets and food composition, temperature, and gender/age, to explore the key affecting environmental factors on Tibetan macaques’ activity rhythms and time budgets. At the same time, the results of this study were compared with the previous studies on other Macaca species to explore the effects of social factors (age and sex) and environmental factors on the activity rhythm and time budgets of Tibetan macaques.

## 2. Materials and Methods

### 2.1. Study Site and Subjects

**Figure 1:**
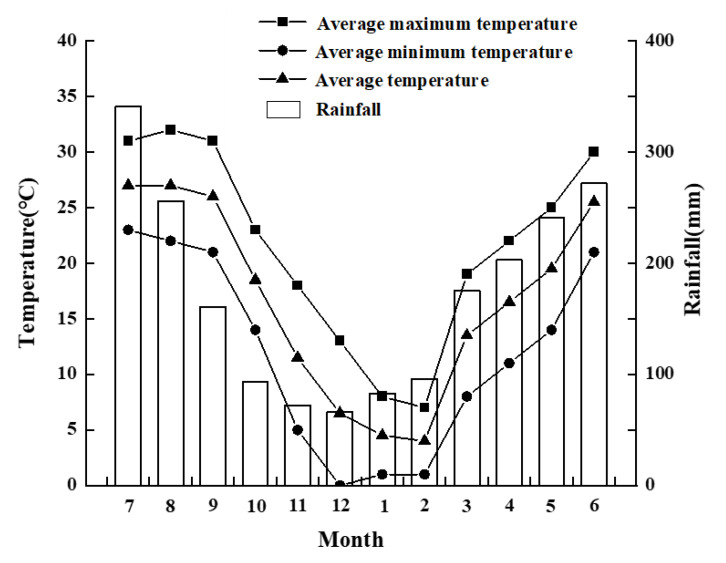
Monthly rainfall and maximum, minimum, and average temperatures at Tianhu Mountain County Nature Reserve.

This study was conducted in Huangshan District, Huangshan City, Anhui Province (30°12′ N, 118°27′ E), which includes the Tanjiaqiao and Tangkou towns (parts of the Huangshan Scenic Area), where the primate research team of Anhui University established a field research center in 2018. The local climate is mainly subtropical monsoon, with the lowest elevation of approximately 500 m a.s.l. and the highest elevation of approximately 1300 m a.s.l. The study area consists of several mountains, including Maota, Donggualing, Tianhu, Sukeng, Hongjialing, Huangtanling, and some non-named mountains in the eastern part of the Huangshan Mountains [26]. Huangshan mountains are full of ravines, and the shady and sunny cliffs are subject to a large difference in solar radiation intensity, whereas the local terrain has the greatest influence on its climate. The study site has a mid-latitude subtropical climate with an annual mean temperature of 16.7 °C, a maximum temperature of 37 °C, a minimum temperature of −6 °C, with an annual rainfall of 2059 mm. Considering the stable mean temperature to be >22 °C in summer, <10 °C in winter, and 10 °C–22 °C in spring and autumn, the four seasons of the study area were classified as spring (March–May), summer (June–September), autumn (October–November), and winter (December–February) [27] (Figure 1).

Tibetan macaques live in groups of males and females. They mainly rely on female kinship to maintain group stability and have a distinct matrilineal social group with a strict social hierarchy. In Oct 2018, one of the members of the Anhui University primate research team (Bo Li) discovered an un-provisioned group of Tibetan macaques near Niejiashan, Tanjiaqiao Town, and since then, we have been conducting studies on this un-provisioned Tibetan macaques. In late 2019, follow-up studies revealed large groups of macaques in the wild, consisting of as many as 91 macaques. In early 2020, these large groups were eventually naturally divided into two smaller groups. The research team began to follow up on the smaller groups in Tianhu Mountain County Nature Reserve (Named TianhuII Group (THII)).

### 2.2. Behavioral and Environmental Factors

Due to the complicated environmental conditions, we could not identify each individual. Therefore, the macaques were only divided into three sex-age groups: adult males, adult females, and immature. We followed the Tianhu group when encountered. We identified the subjects as an old female with a left-hand disability, an adult male with only a left leg, and other individuals with easily recognizable facial features such as α male and female eyebrow shape. The average distance between the group and the observers throughout this study was more than 100 m (Through visual inspection and experience). Therefore, the instantaneous scanning sampling method was used to collect relevant behavioral data. From July 2021 to June 2022, the macaques were observed for 3–7 days per month for a total of 51 days.

When scanning the macaques, we used binoculars. Behavioral sampling usually began when the macaques were first spotted. If the nocturnal site of the previous day could be determined, behavioral sampling began at 06:00 the next day, and observations continued until the macaques entered the nocturnal site. Each scan lasted for 5 min, and sampling intervals were 5 min to ensure relative independence between samples. If the macaques disappeared from the observer’s view, sampling was stopped until the macaques were found again. Each scan sampling started from the left to the right. This was to avoid duplicate recordings. While recording the behavior, we also recorded the macaques’ age. We also recorded the parts of plants, named leaf, flower, fruit, and others, when the macaque was foraging. At each sampling, as many macaques as possible were sampled by changing the observation location to ensure that sampling was not biased toward macaques of a particular sex-age group. Referring to Li et al. (2022) [28], the various behaviors were defined as follows:

1. Resting: an individual sitting with its hips on the ground and its only movement being head rotation, lying on the ground, or being stationary for an extended period (>5 s) during other behaviors such as moving and foraging;
2. Moving: any behavior that causes a change in the individual’s position, such as walking, climbing, jumping, and running (>5 s);
3. Foraging: refers to a set of behaviors that include searching for food, handling food, and finally eating food;
4. Grooming: refers to the grooming behavior of two or more individuals with each other;
5. Others: All other behaviors that occur in animals other than those mentioned above and include social behaviors such as climbing, bridging, playing, and mating.

In addition, for direct observation, we used infrared cameras (ULM-4). We divided the study site into 1 × 1 km grids, and we randomly placed infrared cameras within the grids. Each camera was spaced no less than 300 m apart. The cameras were mainly deployed in broad-leaved forests and mixed coniferous forests at an altitude of 500–1300 m a.s.l. and the locations with high traces of macaques activity (feces, tracks, water sources, etc.) were selected for camera installation. The camera’s height was 0.4–0.5 m above the ground, and the camera was adjusted according to the terrain so that the lens was parallel to the ground. The shooting mode was 24 h continuous operation, and the shortest interval between two photos was 0.8 s; a 15 s video was obtained after each trigger [26]. The infrared camera can quickly send the images to our mobile phones to quickly find the monkeys. We checked the camera every 3–6 days to confirm that it was still working (some large animal could have damaged it).

### 2.3. Data Analyses

The activity time budget was calculated as described by Di Fiore and Rodman (2001) [18]. Each scan sample was considered an independent sample, and the ratio of the number of individuals with a specific behavior type to the total number of individuals observed in the scan sample was used to express the proportion of time spent on this behavior type; then, the data from the scan samples in each hour were averaged to calculate the hourly activity time budget; subsequently, the hourly activity time budget was used as the primary calculation unit to measure. Finally, the activity time budget for each month was used as the basic unit of calculation. The monthly average was then used to calculate the activity time budget for different seasons and the whole year. Daily activity rhythms were expressed as the average of the percentages of the main activity types (resting, moving, foraging, grooming, and other) in each period (1 h). We considered each foraging observation as an independent sample when calculating food proportions. We calculated the proportion of different plant parts for the food composition.

Statistical analyses were performed using the one-way analysis of variance test for differences in activity time budgets between periods in the daily activity rhythm. The data were tested for variance chi-square before using this analysis method. The data that did not meet the requirements were transformed by absolute value (numexpr). It was made to comply with the requirements before analysis.; chi-square test was used to compare seasonal variations in various activities; Mann–Whitney *U* test was used to compare differences between two independent samples; and Spearman’s Rank Correlation Test was used to test the correlation between the variables. All data were processed and analyzed using Microsoft Excel 2019 and SPSS 20 for the Windows software platform.

## 3. Results

### 3.1. Daily Activity Rhythm

**Figure 2:**
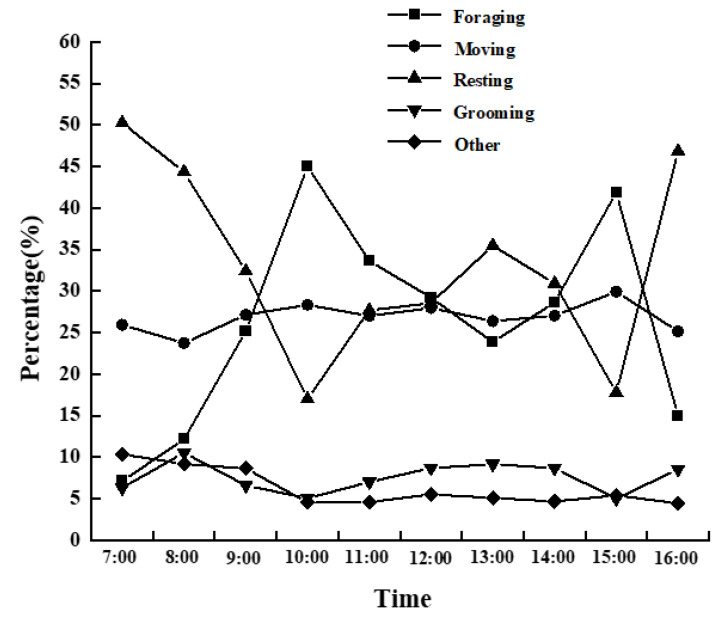
Diurnal activity patterns of Tibetan macaques at Tianhu Mountain County Nature Reserve.

A total of 312 h of follow-up observations were collected throughout the study period, resulting in a total of 1872 scans and an average of 156 scans per month. The results of daily activity rhythms revealed that the proportion of foraging behavior was significantly different in different periods (*F* = 2.703, *df* = 9, *p* < 0.05), mainly manifested by two peaks of foraging in the morning (9:00–10:00) and afternoon (14:00–15:00). Furthermore, the proportion of rest was significantly different between periods (*F* = 3.327, *df* = 9, *p* < 0.05), mainly manifested by a long rest in the morning near the nocturnal site again and a peak of rest at noon (between 12:00 and 13:00) and after 16:00. The movement behavior of the macaques showed an upward trend since leaving the nocturnal site. Still, the proportion of time spent moving did not reach a statistically significant difference (*F* = 0.712, *df* = 9, *p* > 0.05). The proportion of other behavior was significantly different in different periods (*F* = 2.516, *df* = 9, *p* < 0.05). The proportion of time spent grooming did not reach a statistically significant difference (*F* = 1.373, *df* = 9, *p* > 0.05) (Figure 2).

### 3.2. Daily Activity Time Budget and Seasonal Changes

In the daily activity time budget of Tibetan macaques, 31.96% of the time accounted for resting, 28.59% for foraging, 26.96% for moving, 6.90% for grooming, and 5.59% for other behavior (Table 1).

Considering the Tibetan macaques’ daily activity time budget, the proportion of time spent foraging varied from 31.57% (September) to 19.67% (July), moving varied from 36.55% (July) to 13.28% (May), and resting varied from 44.37% (May) to 26.38% (June). Time spent on foraging and moving per month was significantly and negatively correlated with the time spent resting (*r* = –0.874, *n* = 12, *p* < 0.001).

**Figure 3:**
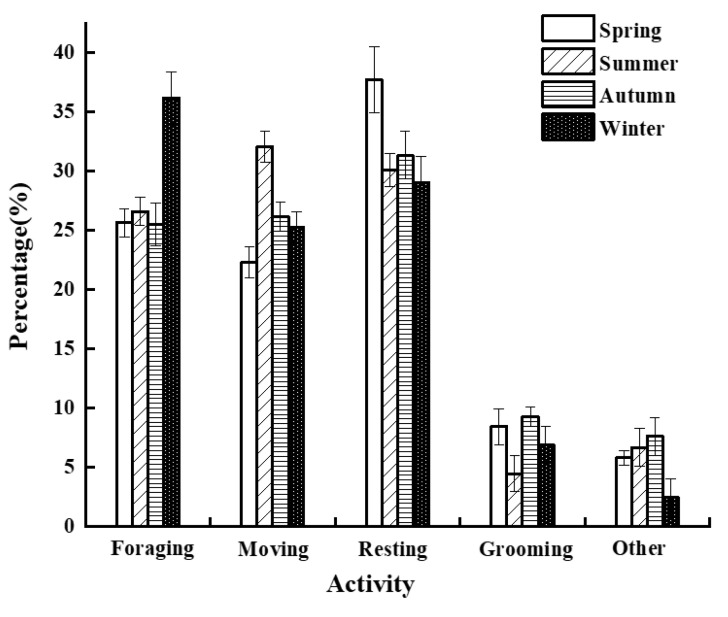
Seasonal variations in the time budgets of various activities.

Additionally, we found that foraging, movement, resting, grooming, and other behaviors were not statistically significant (foraging: *X*^2^ = 6.372, *df* = 3, *p* = 0.095; moving: *X*^2^ = 5.718, *df* = 3, *p* = 0.126; resting: *X*^2^ = 4.032, *df* = 3, *p* = 0.258; grooming: *X*^2^ = 6.295, *df* = 3, *p* = 0.098; and others: *X*^2^ = 5.474, *df* = 3, *p* = 0.140) (Figure 3).

### 3.3. Food Composition, Temperature, and Time Budget

Among the food composition, fruits were the most foraged, consisting of 55.31% of the total foraged food, followed by leaves (21.25%), buds (19.95%), stems (2.29%), flowers (1.18%), and other (0.03%). We compared the monthly food composition ratios considering the time budget for each activity type. We found that the sum proportion of time spent foraging and moving per month was significantly and positively correlated with the sum proportion of fruit and bud foraging (*r* = 0.650, *n* = 12, *p* = 0.022), whereas it was negatively correlated with the proportion of leaf foraging (*r* = –0.692, *n* = 12, *p* = 0.013).

**Figure 4:**
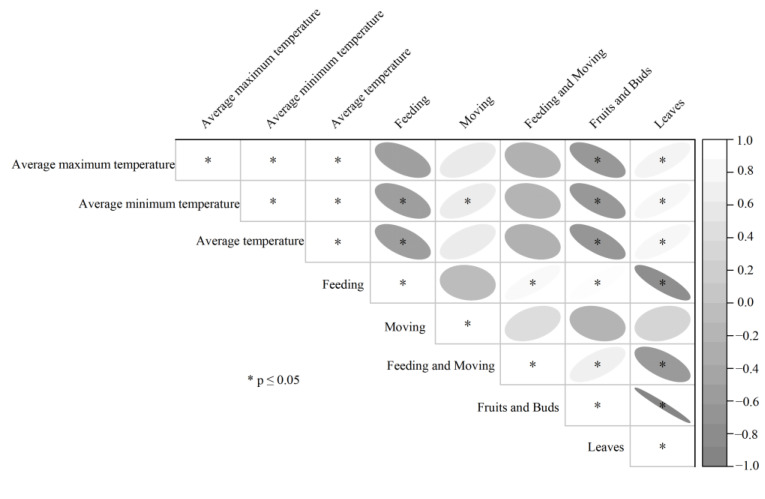
Food, temperature, and time budget correlation.

Some environmental factors also influenced the activity time budget of Tibetan macaques. The relationships between foraging behavior, movement behavior, resting behavior, and the maximum, minimum, and average ambient temperatures and precipitation were analyzed separately. Results showed that the time allocated for foraging behavior was inversely proportional to the average ambient temperature (*r* = –0.599, *n* = 12, *p* = 0.040) and similarly with the average minimum ambient temperature (r = –0.583, *n* = 12, *p* = 0.046), and the time budgeted for movement behavior was positively correlated with the average minimum ambient temperature (*r* = 0.598, *n* = 12, *p* = 0.040) (Figure 4).

### 3.4. Gender/Age and Activity Time Budget

**Figure 5:**
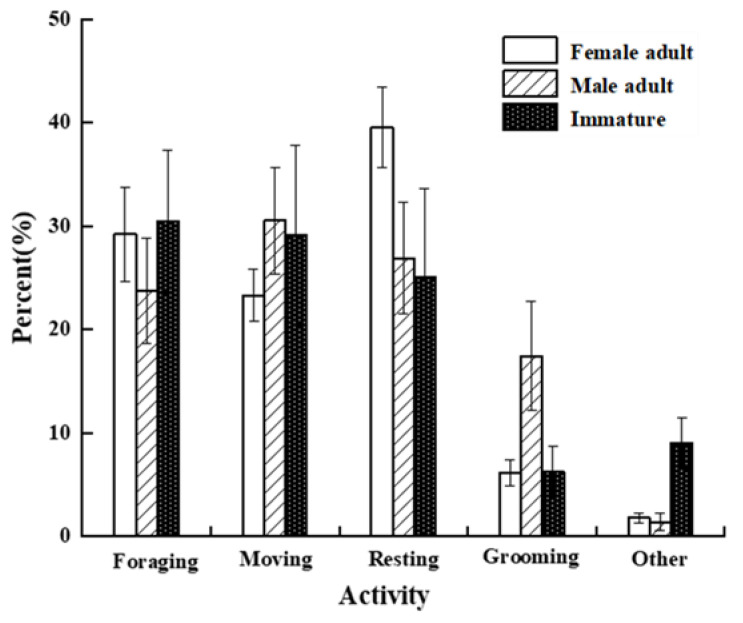
Comparison of time budgets between different sex-age groups.

Significant variability was observed in the activity time budget among individuals of different sex-age groups. The results showed that adult males spent 23.30% of their time moving, which is significantly less than the 30.54% spent by adult females (*Z* = −2.714, *n* = 12, *p* < 0.01) and the 29.14% spent by immatures (*Z* = −2.367, *n* = 12, *p* < 0.05). Furthermore, adult males spent 39.56% of their time resting, which is significantly more than the 26.91% spent by adult females (*Z* = −3.869, *n* = 12, *p* < 0.001) and the 25.10% spent by immatures (*Z* = −4.157, *n* = 12, *p* < 0.001). Additionally, adult females spent 17.45% of their time on grooming behaviors, which is significantly more than the 6.13% spent by adult males (*Z* = −3.695, *n* = 12, *p* < 0.001) and the 6.29% spent by immatures (*Z* = −3.580, *n* = 12, *p* < 0.001). Lastly, immatures spent 9.02% of their time on other behaviors, which is significantly more than the 1.79% spent by adult males (*Z* = −3.523, *n* = 12, *p* < 0.001). *p* < 0.001) and the 1.39% spent by adult females (*Z* = −3.595, *n* = 12, *p* < 0.001) (Figure 5).

## 4. Discussion

### 4.1. Activity Rhythm

In this study, the activity rhythm of Tibetan macaques in the Tianhu Mountain County Nature Reserve was mainly characterized by two foraging peaks, one at 9:00–10:00 and the other at 14:00–15:00, and a resting peak at 12:00–13:00 noon. Similar findings were observed in other primates, such as Hanuman langurs (*Semnopithecus entellus*), Rhesus macaques (*M. mulatta*), and captive mantled guereza (*Colobus guereza*) [29,30,31]. However, not all primates have a clear peak in their daily foraging activity rhythm, for example, Assamese macaques (*M. assamensis*) and Yellow baboons (*Papio cynocephalus*) [32,33]. Assamese macaques, which also belong to the genus *Macaca*, all behaviors did not differ significantly in their daily activity rhythms throughout the year. Foraging activity showed a tendency to increase, with the highest peak occurring in the afternoon. The reason for this phenomenon is that the daily activity rhythms of Assamese macaques vary considerably from season to season and even from day to day, and potential foraging peaks may be masked when all sampling days are analyzed together. A similar situation occurs in yellow baboons living in Kenya. During the dry season, the daily activity rhythms of yellow baboons were similar to those of the whole year, but during the rainy season, three foraging peaks occurred, in the early morning, midday, and late afternoon, but with significant seasonal variability. Therefore, when all daily activity rhythms were combined, there were no significant foraging peaks throughout the year [33]. The activity rhythm of animals in nature is complex. Each animal shows a specific activity pattern to adapt to different living environments [34]. Priimates’ activity rhythm is a long-term adaptation phenomenon, which includes adaptation to abiotic conditions, such as temperature, humidity, and light; ease of access to food; social relationships; interspecific competition; and other biotic conditions of long-term integrated adaptation [35]. Clutton-Brock (1977) pointed out that primates foraging peaks in the morning and afternoon and long rest periods in the middle of the day may be to adapt to temperature fluctuations during the day [29].

In Huangshan mountains, which belong to the Huanggang rocky peaks and forests, the surface rocks’ temperature rises sharply in the hot summer, particularly in the absence of shade, and reaches its peak in the afternoon. Still, the temperature is substantially lower in the thick woods, with the shade of trees. During our study, in the hot summer, Tibetan macaques often hid in places such as clearings, roots, and tree trunks in dense forests to rest away from direct sunlight for 1–2 h. This phenomenon was more pronounced on sunny days. Therefore, we believe that Tibetan macaques may rest for long hours in summer to avoid high temperatures and intense sunlight at noon. By contrast, Tibetan macaques sit in groups on exposed rocks to bask in the sun during the cold winter months, which may be an adaptive strategy to the severe winter cold. The activity rhythms of white-headed langurs are similar [36]. Lawes and Piper propose a different view [37]. They believe that primates eat some fibrous food in the morning, and therefore, they need to take a long rest at noon to digest them. This may also be the reason for the Tibetan macaque’s long midday rest.

### 4.2. Activity Time Budget

Tibetan macaques spent 55.55% of their time foraging and moving and 31.96% of their time resting. Various factors influence the budget of primate activity time, such as habitat quality, climatic factors, and food resources [38,39]. Among them, food resource abundance, quality, and spatial distribution are important factors affecting the time budget [18,40,41]. Wang et al. (2008) studied a group of provisioned Tibetan macaques in the spring, where foraging, wandering, resting, playing, others, and grooming accounted for 14.82%, 5.86%, 59.72%, 0.04%, 2.06%, and 17.50% of the time allocation, respectively. Compared with un-provisioned Tibetan macaques in this study, they need to spend much time searching for food and eating, and hence, they spend much time foraging and moving and more time resting and grooming. In contrast, the provisioned population in this study needed to spend more time on foraging, so resting and grooming behaviors were correspondingly reduced [24].

In the study on Rhesus macaques (*M. mulatta*), the activity time budget was similar to that of macaques inhabiting karst habitats, 62.5% of their time foraging and moving and 29.6% of their time resting. Seth et al. conducted a study on Indian Rhesus macaques (*M.* *mulatta*), revealing that they spend 58% of their time foraging and moving and 35% resting [42]. However, in the study by Zhou et al. on white-headed langurs in Fusui Precious Animal Sanctuary, the results were more varied, with the white-headed langurs spending the most significant proportion of time resting (46.4%) and a proportion of their time foraging and moving (49.3%) [10]. Similarly, Zhou et al. studied Rhesus macaques (*M. mulatta*), which live in a semi-wild state in the Qixing scenic area in Guilin, Guangxi, and found that they spend 39.3% of their time moving and foraging and 41.5% of their time resting (Table 2) [43].

The reason for these differences may be the diet composition. In Tibetan macaques, the proportion of fruits in the diet is 55.31%. By contrast, in Fusui Precious Animal Sanctuary, the proportion of fruits and seeds in the diet of white-headed langurs is only 6%, and the proportion of fibrous food such as leaves is 91% [10]. Furthermore, in the food composition of the macaques living in the Seven Star Scenic Area, we found that they mainly feed on leaves, accounting for 41% of the diet, whereas fruits and seeds account for only 8.5% [43].

In the Huangshan forests, leaves are relatively uniform and abundant; therefore, macaques can easily feed on them. Conversely, the fruits are patchy and not as plentiful as that of leaves, and therefore, macaques have to invest more time and effort to find them [48]. The Celebes crested macaques (*M*. *nigra*) live in the Tangkoko-DuaSudara Nature Reserve. They consume fruits account for 66%and they spend more than half of their time (60%) foraging and moving [44]. For primates whose main diet is leaves, for example, Francois’ langur (*T. francoisi*), leaves account for 70.99%, and fruits and seeds account for only 17.42%. Although their food is more accessible, it is difficult to digest, and therefore, they need less time to forage (18.61%) and more time to rest (51.99%) to ferment, break down, and absorb the nutrients in their food [5,49]. However, not all folivores follow the activity pattern described above. Assamese macaques (*M*. *assamensis*) are typical folivores in the limestone forest; they spent >60% of their activity time moving and foraging, indicating that they are extremely active. This behavior is similar to that of frugivorous macaques. This characteristic could be linked to their bamboo leaf-based diet [47]. Barbary Macaques (*M. sylvanus*) living in the Middle Atlas Mountains of Morocco [50] spent more time foraging and less time foraging when snow coverage was between 1% and 49% and more time foraging at lower temperatures. Tibetan macaques are similar to them. We found that the time they spent on foraging activities was inversely proportional to the average temperature. Macaques response to snow coverage and temperature may represent a strategy to maximize foraging efficiency by attempting to ingest more food at lower temperatures (when the daily energetic requirement for thermoregulation is greater [51]) and when snow is present [52,53]. In our study, a similar strategy was found to be adopted by Tibetan macaques. In winter, they spend more time foraging for food. The Atlas Mountains are affected by extreme weather. The area where the Barbary macaques (*M. sylvanus*) live is covered with snow. Snow coverage significantly reduces access to food sources and forces an increased time for foraging. In addition, when the snow reaches a certain thickness, the food available to the monkeys will be greatly reduced. Several monkeys probably did not survive the severe winter of 2008–2009 for the reasons mentioned above. Compared to the Atlas Mountains, the climate in this study area is more moderate. There is not too much snow cover. And winter food is more abundant such as sweet chink and bamboo shoots. Tibetan macaques can easily obtain sufficient food to cope with the low temperatures in winter. Moving time in Japanese macaques (*M*. *fuscata*) would decrease with decreasing temperature [15]. This is similar to the Tibetan macaques(*M*. *thibetana*). Foraging and moving are both energy-consuming behaviors (compared to resting). At lower temperatures, the benefits of foraging and movement may not be greater than those of resting.

Foraging and ranging patterns of lion-tailed macaques (*M. silenus*) showed that they were predominantly frugivorous and insectivorous evergreen forest species [46]. Fruits and insects are more difficult types of food to obtain compared to leaves. As a result, they spend close to 70% of their time foraging and moving around. In contrast, Tibetan macaques mainly feed on fruits, leaves, and buds. Food acquisition is less difficult compared to the lion-tailed macaques (*M. silenus*). Therefore, Tibetan macaques also spend less time foraging and moving than lion-tailed macaques.

### 4.3. Seasonal Variations

The total foraging and moving time per month was significantly and positively correlated with the sum proportion of fruits and buds and negatively correlated with the proportion of leaves. When environmental resources change, animals can change their activity time budget [22,54]. Norberg and Ake (1977) proposed two models to explain the behavioral strategies adopted by animals in response to environmental resources, namely passive foraging strategies and positive foraging strategies. Passive foraging strategy: When the environment is full of high-quality food, animals will spend more time and effort finding high-quality food. They use this method to achieve a higher net energy income. When high-quality food is scarce, animals spend less time foraging to conserve energy. Positive foraging strategies: When high-quality food is lacking in the environment, animals spend more time and energy searching for food to replenish their energy [55].

In the study by Zhou et al. on white-headed langurs (*T*. *leucocephalus*), the monkeys tended to use an active foraging strategy, spending more time and energy foraging for preferred food items even when they were scarce [10]. However, Rhesus macaques (*M. mulatta*) showed the opposite foraging strategy; they increased foraging time when preferred food was abundant and decreased time when it was scarce [5].

In this study, late summer, early autumn, late winter, and early spring were the seasons during which high-quality food (e.g., yellow wax fruit, sweet chink, and bamboo shoots) was abundant. During this time, macaques increase the time and energy spent on foraging. When preferred food is scarce, macaques reduce the foraging and increase resting time, saving energy and preserving stamina [56]. Therefore, Tibetan macaques prefer to use passive foraging strategies to cope with environmental changes in food resources.

In this study, we found significant differences in the rest time budget. Adult males spend more time resting than immature and adult females do. A possible reason for this is that adult males need to guard their surroundings and protect the group. Therefore, they spend most of their time sitting alone to rest. Adult females usually sit with other individuals. This increases their chances of contact. Therefore, they spend more time grooming than other Macaca species [42]. In addition, grooming each other can reduce tension, which positively affects intragroup stability [57]. Furthermore, grooming creates an alliance between individuals, facilitating the appropriation of resources and mates [58]. Immatures spend 93.66% of their time playing (8.44% of the overall time), whereas adults spend only 8.77% of their time playing (0.14% of the overall time). Immatures may learn how to move efficiently on the ground through playful behavior. Furthermore, play behavior may be used to determine one’s social status in the group [59].

## 5. Conclusions

Primates’ activity rhythms and time budgets are important to understand their behavioral variability and adaptation to seasonal change habitat. In this study we found that Tibetan macaques consists of two foraging peaks (in morning and afternoon) and one resting peak (in midday). They spend 31.96% forresting, 28.59% for foraging, 26.96% for moving, 6.90% for grooming, and 5.59% for others. Temperature influenced Tibetan macaques’ activity time budget. The foraging behavior was inversely proportional to the temperature and similarly, the time budgeted for movement behavior was positively correlated with the average minimum temperature. In addition, we also found that there were significantly different in sex-age groups on the activity time budget, which showed that social factors may also influence the primates’ activity rhythms and time budgets. Because we could not be accurately identified each individuals in this study, it was not clear which social factors may influenced Tibetan macaques’ activity rhythms and time budgets. We hoped that more future studies should focuse on the un-provisioned (accurate identification of individual) Tibetan macaque group.

## Figures and Tables

**Table 1:** Monthly and annual time budgets as a percentage of time spent on each activity type.

Month	Resting	Foraging	Moving	Grooming	Other
July	31.78	19.67	36.55	4.55	7.45
August	35.39	23.66	31.02	4.32	5.61
September	26.96	31.57	28.27	3.74	9.46
October	27.27	27.42	30.14	9.45	5.72
November	35.43	23.62	22.21	9.14	9.59
December	26.86	33.59	26.46	8.81	4.28
January	29.99	37.56	24.05	6.54	1.86
February	30.32	37.46	25.37	5.54	1.32
March	34.16	27.19	30.22	4.51	3.92
April	34.65	28.90	23.50	10.59	2.36
May	44.37	20.90	13.28	10.27	11.18
June	26.38	31.53	32.40	5.40	4.29
Mean	5.23	5.97	5.98	2.56	3.23
Standard deviation	31.96	28.59	26.96	6.90	5.59

**Table 2:** Comparison of activity rhythms and time budgets of macaque species in the wild.

Species	Foraging	Moving	Resting	Grooming	Other	Playing	Research Location	Survival Environment	Reference
Tibetan macaques (*M. thibetana*)	28.59	26.96	31.96	6.90	5.59	*	Tianhu Mountain County Nature Reserve, Anhui Province, China	Granite body peak forest landform, subtropical evergreen broad-leaved forest.	This study
Tibetan macaques (*M. thibetana*)	14.82	5.86	59.72	17.50	2.06	0.04	Floating Creek Monkey Valley, Anhui Province, China	Granite body peak forest landform, subtropical evergreen broad-leaved forest.	Wang et al., 2008 [24]
Rhesus macaques (*M. mulatta*)	37.30	25.20	29.60	2.20	0.20	5.50	Nonggang Nature Reserve, Guangxi Province, China	Limestone mountainous terrain, evergreen seasonal rainforest	Tang et al., 2011 [9]
Rhesus macaques (*M. mulatta*)	11.60	27.80	41.50	7.00	*	12.10	Qixing scenic area, Guangxi Province, China	Karst landform	Zhou et al., 2009 [43]
Celebes crested macaques (*M. nigra*) Group1	34.10	18.30	28.90	18.70	*	*	Tangkoko-DuaSudara Nature Reserve (TDS) on the northern peninsula of Sulawesi, Indonesia	lowland rainforest	O’ Brien and Kinnaird, 1997 [44]
Celebes crested macaques (*M. nigra*) Group2	38.60	25.70	12.60	23.10	*	*			
Celebes crested macaques (*M. nigra*) Group3	36.00	23.50	17.00	23.50	*	*			
Rhesus macaques (*M. mulatta*)	36.20	11.00	36.80	11.00	0.60	4.40	Bangladesh	a habitat surrounding village	Firoj Jaman and Michael 2013 [45]
Rhesus macaques (*M. mulatta*)	22.40	10.80	46.10	16.50	1.10	3.10		a habitat surrounding urban	
Japanese macaques (*M. fuscata*)	38.00	16.00	32.00	14.00	*	*	western area of Yakushima, Japan	Coniferous Forest	Hanya, 2004 [15]
Lion-Tailed Macaques (*M. silenus*)	54.50	15.00	27.00	2.40	1.10	*	Anamalai Wildlife Sanctuary, Tamil Nadu, India	evergreen forests	Kurup and Kumar, 1993 [46]
Assamese Macaques (*M. assamensis*)	32.70	28.60	28.60	8.00	0.40	1.70	Nonggang National Nature Reserve, Guangxi Province, China	Limestone Forest	Li et al., 2020 [47]

(“*” means that this behavior was not counted separately in the study).

## Data Availability

Not applicable.
